# A numerical analysis of a magnetocaloric refrigerator with a 16-layer regenerator

**DOI:** 10.1038/s41598-017-14406-9

**Published:** 2017-10-25

**Authors:** Mingkan Zhang, Omar Abdelaziz, Ayyoub M. Momen, Ahmad Abu-Heiba

**Affiliations:** Energy and Transportation Science Division, Oak Ridge National Laboratory, Oak Ridge, TN 37831 USA

## Abstract

A numerical analysis was conducted to study a room temperature magnetocaloric refrigerator with a 16-layer parallel plates active magnetic regenerator (AMR). Sixteen layers of LaFeMnSiH having different Curie temperatures were employed as magnetocaloric material (MCM) in the regenerator. Measured properties data was used. A transient one dimensional (1D) model was employed, in which a unique numerical method was developed to significantly accelerate the simulation speed of the multi-layer AMR system. As a result, the computation speed of a multi-layer AMR case was very close to the single-layer configuration. The performance of the 16-layer AMR system in different frequencies and utilizations has been investigated using this model. To optimize the layer length distribution of the 16-layer MCMs in the regenerator, a set of 137 simulations with different MCM distributions based on the Design of Experiments (DoE) method was conducted and the results were analyzed. The results show that the 16-layer AMR system can operate up to 84% of Carnot cycle COP at a temperature span of 41 K, which cannot be obtained using an AMR with fewer layers. The DoE results indicate that for a 16-layer AMR system, the uniform distribution is very close to the optimized design.

## Introduction

The magnetocaloric effect (MCE) is a phenomenon where a temperature change of a magnetocaloric material is caused by exposing the material to a changing magnetic field. A cooling technology was developed based on MCE, however, studies of this technology were limited to the maximum adiabatic temperature change of 5 K due to the MCE limitations of most MCMs at moderate magnetic fields (up to 1.5 T)^1^. Barclay and Steyert^2^ introduced the AMR concept to address the problem, in which a regenerative cycle is applied to create temperature spans comparable to conventional cooling systems. Following their work, investigations in the magnetic refrigeration community focused on the AMR as it provided a methodology to realize an alternative refrigeration technology with significant potential energy savings compared to conventional vapor compression refrigeration technology at room temperature. A large number of experimental and numerical studies have been conducted on AMR research, which have been illustrated in the review papers^3–10^.

The research in magnetocaloric refrigeration in the past decades led to an increased interest in the search for new MCMs with high MCE. Initially, the materials used in the AMR studies focused on the ones with second-order phase transition, such as Gd and its alloys based on rare-earth materials (GdTb, GdEr, GdDy, etc.). However, the commercial use of those materials is limited not only by their relative low MCE, but also by their relatively high cost^11,12^. The major breakthrough occurred in 1997 when the giant MCE in a GdSiGe alloy was discovered by Pecharsky and Gschneidner^13^, which had twice the magnetic entropy change compared to that of Gd. The discovery of the giant MCE, which is due to the first-order phase transition (FOPT), stimulated the research on MCMs worldwide. This was followed by the discoveries of a number of other alloys with FOPT and pronounced MCE, based on MnAsSb^14^, MnFe(P,As)^15^, MnFe(P,Si)^15^, La(Fe,Mn,Si)H^16^ and LaFeSi(Co,H)^17,18^. Among the newly discovered materials, the LaFeSi based ones are very promising candidates as the working materials for magnetic refrigeration at room temperature. Even though their adiabatic temperature change is about the same as Gd, LaFeSi based MCMs have higher entropy change. They also have small hysteresis, and relatively low price (small amount of La usage)^19^.

However, disadvantages of FOPT exist for AMR implementation. One of its fatal disadvantages is the relatively narrow temperature range of the MCE which tremendously limits the temperature span of the AMR using FOPT materials as MCM. In order to provide the largest temperature span across the AMR, or the largest MCE in the entire AMR, several different MCMs with different Curie temperatures must be layered along its length. These materials can be synthetized by adding additional elements, such as Co or H, to the base materials (e.g. LaFeSi). The Curie temperature and the MCE of the synthesized material can be tuned by controlling the quantity of the added elements. As reported by Liu *et al*.^20^, ternary La(Fe,Si)_13_ alloys have a Curie temperature around 200 K. By adding a fourth element, such as Co or H, the Curie temperature is able to increase up to room temperature. Moreover, by controlling the quantity of additional elements, the Curie temperature can be tuned to cover a wide temperature range.

Due to the necessity of layered materials with different Curie temperatures in AMR, it is desired to study the AMR with multi-layer MCMs. Currently, available literature on the multi-layer AMR is far less than that on the single layer AMR literatures. In 1997, Tsukagoshi *et al*.^21^ reported the refrigeration performance improvement in their experiments by using a 3-layer AMR. Richard *et al*.^22^ conducted a series of experiments to compare the single-layer and multi-layer AMRs, in which the two-layer regenerator was found to produce a higher temperature span and more cooling power than the single material regenerator under certain operating conditions. Rowe and Tura^23^ extended the work from Richard *et al*.^22^ to a three-material layered AMR using Gd based materials. Recently, Teyber *et al*.^24^ reported on their two-layer AMR system using Gd based materials. They discovered that for the no-load conditions, the largest performance improvements were observed in the two-layer regenerators.

With the discovery of the giant MCE, studies turned to FOPT material based multi-layer regenerator. Engelbrecht^25^ experimentally compared four AMRs using three different materials (Gd, La(Fe,Co,Si)_13_ and LCS) including a two-layer regenerator made of La(Fe,Co,Si)_13_ compounds. He concluded that multi-layer regenerator presents a better efficiency than the single-layer. Similar experimental research was conducted by Legait *et al*.^26^ by comparing four AMRs using three different materials (Pr_0.65_Sr_0.35_MnO_3_, La(FeCo)_13−x_Si_x_ and Gd) including a four-layer regenerator. They also observed that the layered regenerator presents a better efficiency than the single layer. Tusek *et al*.^27^ experimentally compared the performance of the AMRs in room temperature magnetic refrigerators using multi-layer LaFeCoSi and single-layer Gd. They tested 2-, 4- and 7-layer LaFeCoSi AMRs and revealed that multi-layer LaFeCoSi AMRs provide higher cooling power than single layered Gd AMR. In their study, it can be concluded that the 7 and 4-layer AMRs exhibited very similar performance, which is mostly due to the relatively poor heat-transfer properties and the heat losses to the surroundings. Most recently, Govindappa *et al*.^28^ conducted an experimental investigation of FOPT material based multilayer AMR, in which 3-layer, 6-layer and 8-layer AMRs were studied. They discovered that it is necessary to have enough MCM length in every layer as the number of layers increases (e.g. 8-layer) to enable all layers to be active.

In addition to experimental studies, a few numerical studies are reported in the literature. Aprea *et al*.^29^ conducted a numerical analysis of multi-layer (up to 6) AMR with Gd_x_Tb_1-x_ and Gd_x_Dy_1−x_ over the temperature range of 275–295 K and 260–280 K, respectively. However, the MCMs limited the working temperature range to non-room temperature. Monfared and Palm^30^ numerically studied a 6-layer packed bed regenerator using Gd based MCMs and optimized the design to obtain maximum performance. Hsieh *et al*.^31^ and Cararo *et al*.^32^ modeled 2-layer and 3-layer AMRs for a room temperature refrigeration also using Gd-based materials as MCMs. Kamran *et al*.^33^ conducted a performance optimization of a layered AMR with up to four different MCMs, suggesting using a smaller Curie temperature difference between the adjacent MCM layers in order to gain higher COP/capacity, which is a rare numerical study using FOPT materials as MCM (LaFe_13−x−y_Co_x_Si_y_).

One of the most significant barriers to modeling layered AMR systems is lack of experimental data for the MCM properties. In addition, typical simulation codes suffer from significant increase in computational resources required (processor and memory) as the number of MCM layers increases. Therefore, previous modeling using properties directly from experiments only addresses AMRs with a few layers (up to 6). Although Lei *et al*.^34^ and Lei *et al*.^35^ reported their modeling of layered AMR with up to 60 layers, the properties of different MCM layers were approximated by shifting experimental data obtained for one layer according to Curie temperature. By doing so, there was not much increase in computational resources comparing to the single layer case. In present work, a 16-layer AMR was numerically studied. Numerical studies highly depend on experimental data, e.g. MCM properties, so 16 is the maximum number of layers in this paper due to the property data limitation. The MCMs are LaFeMnSiH alloys, and the property data was derived from the experimental measurements provided by VACUUMSCHMELZE GmbH & Co. KG. The model presented in this paper was tailored to accelerate the simulation speed, which significantly decreased the processing time for a multi-layer AMR (6 times faster compared to the typical simulation codes) such that it approached the speed of a single-layer AMR simulation. Based on the model, a performance study was conducted to investigate the effects of frequency and utilization on the cooling power and COP of the multi-layer AMR. Then a DoE was employed to optimize the lengths of each of the 16 MCM layers.

## Numerical Method and Verification

**Figure 1:**
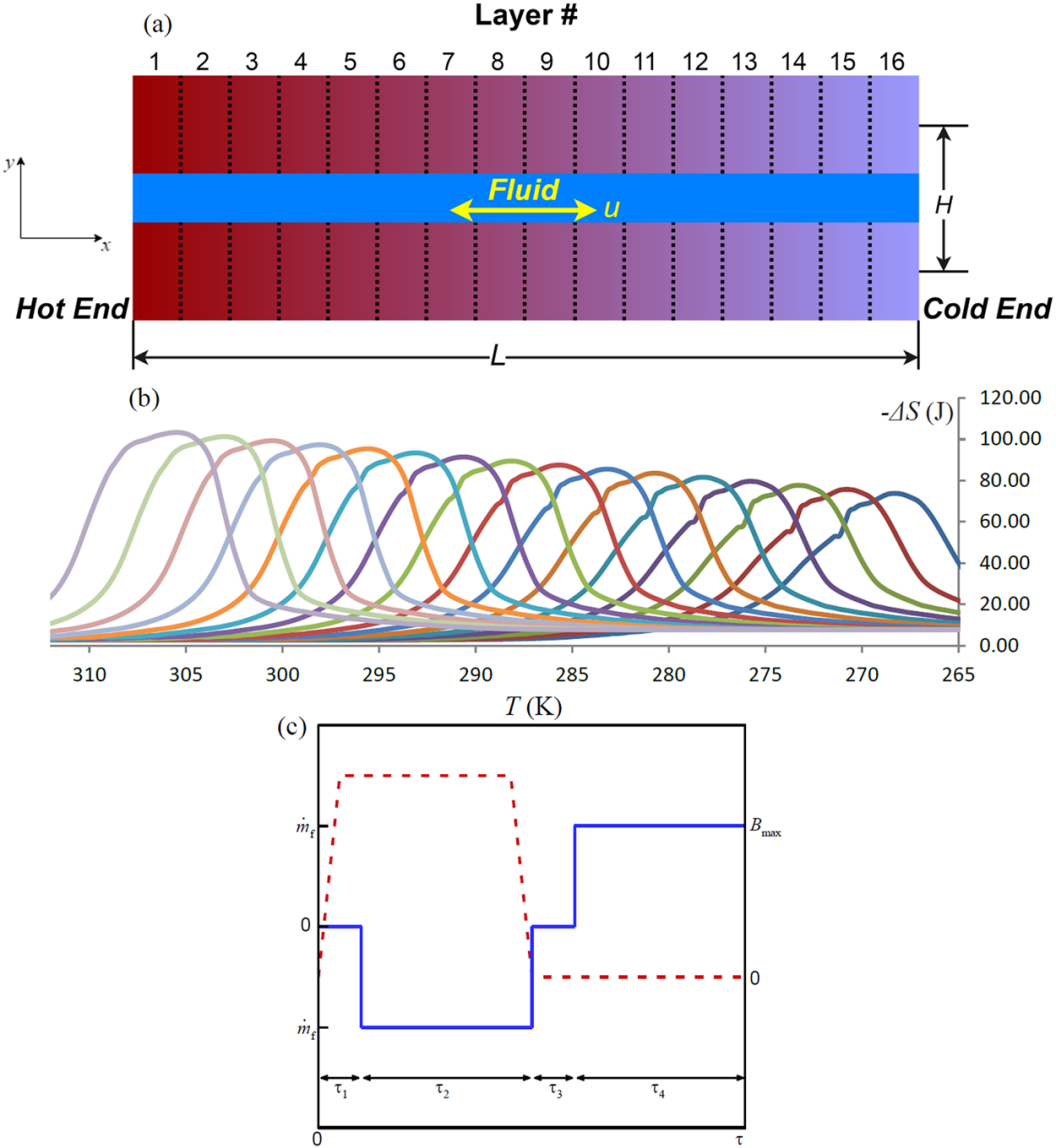
Schematic view of the layered AMR (**a**) with 16 layers entropy changes with temperature (**b**). (**c**) represents the variation of the flow rate (solid line) and the magnetic field (dashed line) during the four steps of the AMR refrigeration cycle, in which *τ*_1_ = *τ*_3_ = 0.1*τ*,and *τ*_2_ = *τ*_4_ = 0.4*τ*. The ramp of the magnetic field lasts 0.5*τ*_1._

The numerical simulations of the 16-layer magnetocaloric parallel plates regenerator are based on layers LaFeMnSiH with different Curie temperatures and a water/glycol mixture. The magnetic refrigerator is comprised of parallel plates with 200 mm as length *L*, 0.75 mm as height *H*, and a unit length width as shown in Fig. 1(a). *x* and *y* are the direction of fluid flow and the direction perpendicular to the fluid flow, respectively. Figure 1(b) shows the entropy change with temperature for each of the 16 MCMs used in the model when the applied magnetic field is varied from 0 to 1.6 T, which also reveals the different Curie temperatures for the MCM.

The effect of plate orientation with respect to external magnetic field on the magnetization is not considered for simplicity. The porosity *ε* in present work is 1/3. The cold end temperature *T*_*C*_ and hot end temperature *T*_*H*_ are fixed at 266 K (−7.15 °C) and 307 K (33.85 °C), based on the Curie temperature of MCM materials at the two ends, so the temperature span in present work is fixed at 41 K. The maximum applied magnetic field *B*_max_ is 1.5 T. The density and thermal conductivity of MCM are 7000 kg/m^3^ and 10 W/(m⋅K), respectively, as reported by the MCM provider. Therefore, the total mass of MCM in one plate is 1.05 kg. The properties of the water/glycol mixture are obtained from^36^ as a function of temperature except the density, since the variance of fluid density has a trivial effect on the results. Similar to the single-layer AMR refrigeration system, the refrigeration cycle with 16-layer parallel plates regenerator also consists of four steps, which are magnetization, cold-to-hot blow, demagnetization, and hot-to-cold blow. The details of the cycle can be found in the literature^37^ and will not described here. Figure 1(c) shows the variation of the fluid mass flow and magnetic field during the refrigeration cycle.

In order to analyze and optimize the magnetic refrigerator system with a multi-layer regenerator, a 1D numerical model was employed, which applies the first law of Thermodynamics to the MCM plates and the heat transfer fluid. The governing equations that describe the heat transfer in MCM plates and heat transfer fluid are shown in Equations (1) and (2), respectively, which are coupled by a convective heat transfer term. This mathematical model has been widely used and validated by different investigators^30,37–39^.

1 $${h}_{sf}a({T}_{f}-{T}_{s})+{k}_{e,s}\frac{{\partial }^{2}{T}_{s}}{\partial {x}^{2}}=(1-\varepsilon ){\rho }_{s}{T}_{s}{(\frac{\partial s}{\partial B})}_{T}\frac{\partial B}{\partial t}+(1-\varepsilon ){\rho }_{s}{c}_{s}\frac{\partial {T}_{s}}{\partial t},$$

2 $${k}_{e,f}\frac{{\partial }^{2}{T}_{f}}{\partial {x}^{2}}-{h}_{sf}a({T}_{f}-{T}_{s})+\frac{dp}{\partial x}u=\varepsilon {\rho }_{f}{c}_{p,f}\frac{\partial {T}_{f}}{\partial t}+{\rho }_{f}u{c}_{f}\frac{\partial {T}_{f}}{\partial x},$$

In these two equations, *T*, *k*_*e*_, *ρ*, and *c* denote temperature, effective thermal conductivity, density, and specific heat, respectively, while subscripts *s* and *f* indicate MCM and fluid respectively. *a*, *ε*, *s*, *B*, *p* and *u* are volume-specific surface area, porosity, MCM entropy, applied magnetic field, fluid pressure, and fluid velocity, respectively. The *k*_*e,s*_ and *k*_*e,f*_ are defined as Engelbrecht suggested^37^, based on the axial dispersion for a fluid flowing between two infinite plates from Beard^40^,

3 $${k}_{e,s}={k}_{f}(\frac{{k}_{s}}{{k}_{f}}(1-\varepsilon )+\varepsilon ),$$

4 $${k}_{e,f}=\frac{{k}_{f}}{210}{(\frac{PrRe}{2})}^{2},$$

where *k*_*s*_ and *k*_*f*_ are the thermal conductivities of MCM and fluid, respectively. *Pr* and *Re* are the Prandtl Number and Reynolds Number, respectively. *k*_*e,s*_ relates the actual rate of axial conduction through the composite MCM/fluid matrix in the absence of fluid flow to the rate of conduction heat transfer that would be experienced by a comparable solid piece of material. *k*_*e,f*_ considers an augmentation of the thermal conductivity in the fluid due to the eddy mixing of the fluid that occurs when fluid flows^37^.

In Equations (1) and (2), the MCM entropy and specific heat vary with temperature as well as applied magnetic field. However, these two parameters are not independent, i.e. the specific heat of MCM can be calculated from entropy variation with temperature, *c*_*s*_ = *T*_*s*_(∂*s*/∂*T*_*s*_). Therefore, for the 16 MCMs with different Curies temperatures, 16 different sets of *s* property data have to be utilized, which are from the experimental measurements provided by the material provider.

The convection heat transfer coefficient, *h*_*sf*_, is calculated from correlations for the flow between parallel plates^41^,

5 $${h}_{sf}=N{u}_{f}{k}_{f}/{d}_{h},$$

where *d*_*h*_ and *Nu*_*f*_ are the hydraulic diameter and Nusselt number. The Nusselt number is calculated as

6 $$N{u}_{1}={(7.541+{(1.841G{z}^{1/3})}^{3.592})}^{1/3.592},$$

7 $$N{u}_{f}=N{u}_{1}-\frac{1.841}{3}G{z}^{1/3}{(\frac{1.841G{z}^{1/3}}{N{u}_{1}})}^{2.592},$$

where *Gz* is the Graetz number in the regenerator, which is defined as

8 $$Gz=PrRe\frac{{d}_{h}}{{x}_{f}},$$

where the position, *x*_*f*_, is measured from the fluid inlet.

The pressure drop is calculated from Equation (9)^42^,

9 $$dp/dx=f{f}_{f}{\rho }_{f}{u}^{2}/(2{d}_{h}).$$

The friction factor, which includes the viscous developing region of the flow, is shown in Equation (10).

10 $$f{f}_{f}=\frac{96}{Re}.$$

The boundary conditions are shown in Table 1.

**Table 1:** List of Boundary Conditions.

Flow Direction	Hot End to Cold End	Cold End to Hot End
Fluid/Hot End	T_f_ = T_H_	∂T_f_/∂x = 0
Fluid/Cold End	∂T_f_/∂x = 0	T_f_ = T_C_
Solid/Both Ends	∂T_s_/∂x = 0	∂T_s_/∂x = 0

The cooling capacity, rejected heat, COP, and utilization, are calculated using the following equations,

11 $${Q}_{c}=\frac{1}{\tau }\,{\int }_{0}^{{\tau }_{f}}{\dot{m}}_{f}{c}_{f}({T}_{C}-{T}_{f}(x=L))dt,$$

12 $${Q}_{h}=\frac{1}{\tau }\,{\int }_{0}^{{\tau }_{f}}{\dot{m}}_{f}{c}_{f}({T}_{f}(x=0)-{T}_{H})dt,$$

13 $${\dot{W}}_{mag}=({Q}_{h}-{Q}_{c}),$$

14 $${\dot{W}}_{pump}=\frac{1}{\tau }{\int }_{0}^{\tau }|\frac{{\dot{m}}_{f}dp}{{\rho }_{f}}|dt,$$

15 $${\rm{COP}}=\,{Q}_{c}/({\dot{W}}_{mag}+{\dot{W}}_{pump}),$$

16 $$U=\frac{{\dot{m}}_{f}{\tau }_{f}{c}_{f}}{{m}_{s}{c}_{s}},$$

17 $${\bar{Q}}_{c}=\frac{{Q}_{c}}{{m}_{r}},$$

In the above equations, *τ* is the cycle time, which is the multiplicative inverse of frequency *f*. *τ*_*f*_ is the time for cold flow and hot flow, since they are identical in present work. ${\dot{m}}_{f}$ is the mass flow rate. ${\dot{W}}_{pump}$ is the pumping power and calculated using the pressure drop and the flow rate. Utilization is the ratio between the heat capacity of the fluid and that of the magnetocaloric material during a single fluid flow period^43^. The cooling power density (${\bar{Q}}_{c}$) is defined as the generated cooling power by unit mass of MCM.

**Figure 2:**
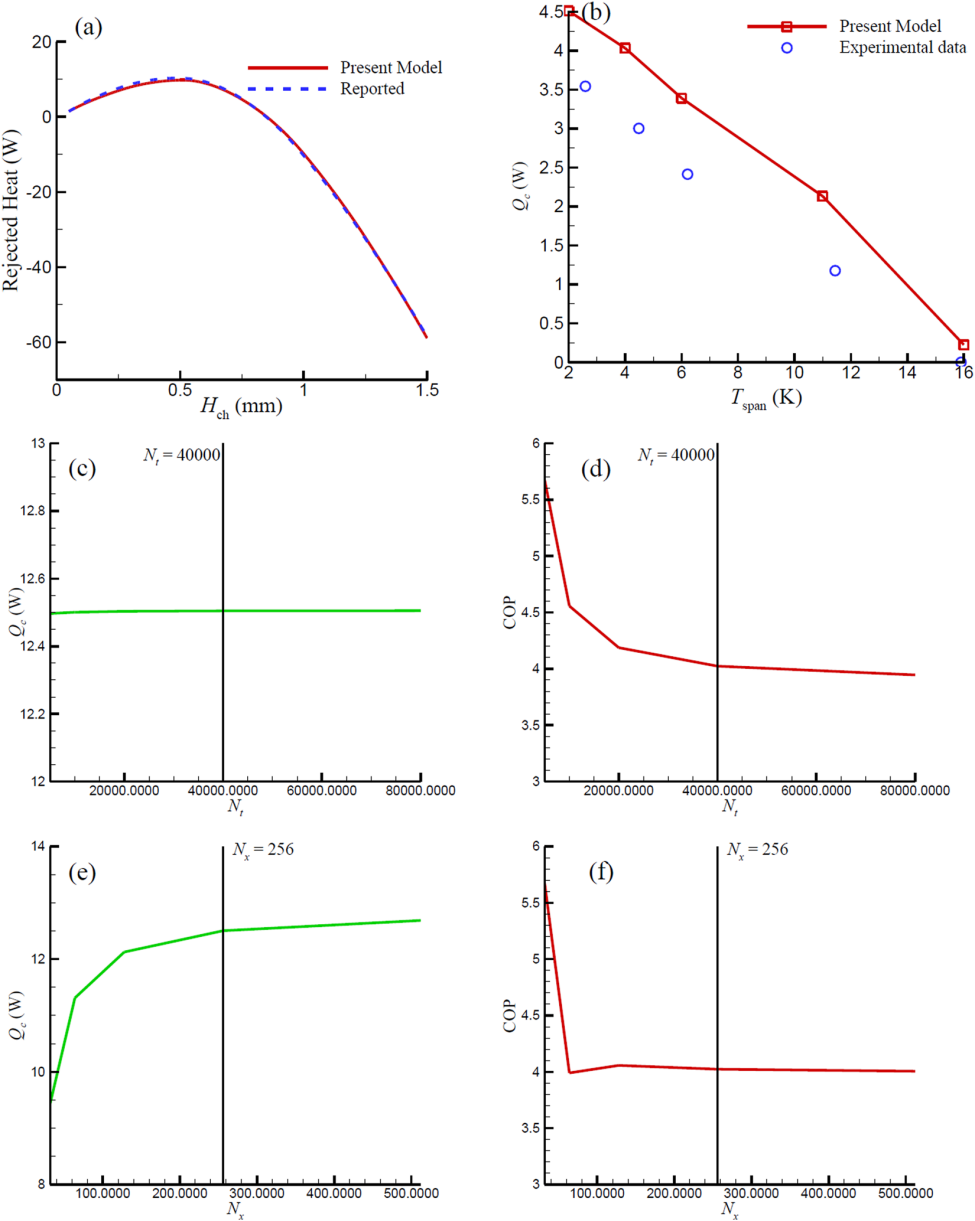
Comparisons between numerical results from Petersen *et al*.^44^ and present model on rejected heat (**a**), and Engelbrecht *et al*. on *Q*_*c*_ (**b**)^45^. COP and *Q*_*c*_ change with time step and mesh size when *N*_*x*_ = 256 ((**c**) and (**d**)) and *N*_*t*_ = 40000 ((**e**) and (**f**)).

The 1D model was implemented in MATLAB^®^. The MATLAB^®^ code is based on^37^, which starts from an initial temperature distribution and terminates after a cyclical steady state has been achieved. Steady state is defined as when the relative change in the energy (fluid energy + regenerator energy) of the regenerator from cycle to cycle is less than a specified tolerance. To verify the developed model, simulation of a single-layer AMR made of Gd using water as the heat transfer fluid with published results from Petersen *et al*.^44^ (numerical results) were compared, since Petersen *et al*.^44^ provide the comprehensive parameters in their paper to benefit the comparison. The comparisons in Fig. 2(a) show a good agreement between each other. Moreover, a comparison was also conducted against experimental data from Engelbrecht *et al*.^45^. As shown in Fig. 2(b), although, there is an over-prediction of *Q*_*c*_ from our numerical model due to the lack of external losses.The single-layer code needs to be extended for multi-layer AMRs. Extension of the original single-layer model to multi-layer suffered from excessively long computation time. The speed issue may be insignificant if only four or six layers are considered. However, for a 16-layer AMR, the increase in computational time makes the model undesirable, e.g. the time required for a 16-layer case using this model running 10 cycles was 10809 seconds, while a one-layer case under the same conditions needed only 1804 seconds. In the simulation, the MCM properties (entropy and specific heat) are preprocessed as matrices in MATLAB that are used as lookup tables, so for multi-layer AMR, every MCM layer has two property matrices. The property matrices need to be interpolated to obtain the entropy and specific heat for a specific temperature and magnetic field. It was found that the most time consuming process in the simulation is the interpolation in properties matrices. The time consumption increases significantly with the number of layers as 2*N_layer_ property matrices have to be interpolated every time step in the simulation. It is also found that the calculation speed of the native MATLAB interpolation function is almost independent of the size of matrix, i.e. the time consumption to conduct an interpolation in a large matrix is almost as same as in a small one. This finding was exploited to reduce the simulation computation time. Instead of using multiple matrices for every layer separately, the multi-layer properties were integrated into two large matrices for entropy and specific heat, individually. A mathematical transformation was employed to change the temperature index of different layers, i.e. the temperature index for layer *N* will add N*100 K, so the properties can be identified for each layer in one matrix. As a result, the speed of 16-layer cases has been significantly increases by a factor of 6. As a result, the 16-layer case solved by the new speed improvement model only needs 1867 seconds to run 10 cycles, which is very close to the time needed for single-layer model.

Figure 2(c) to (f) shows the results of the mesh and time independent study, indicating that COP is sensitive to the time step, so a sufficiently small time step is required in order to accurately capture the dynamics of the 16-layer AMR. Additionally, Fig. 2(e) and (f) show that mesh size does not greatly impact the COP and *Q*_*c*_ from *N*_*x*_ = 256 to 512. According to the mesh and time step study, *N*_*t*_ = 40000 (*Δt* = 1/4000 s) and *N*_*x*_ = 256 (*Δx* = 0.78 mm) are chosen as the parameters for an optimal balance between accuracy and simulation speed.

## Results and Discussions

### Performance Analysis of the 16-layer AMR

*Q*_*c*_ and COP for different utilization and frequencies for single layer AMR have been well investigated by the previous work experimentally, e.g. Trevizoli *et al*. and Trevizoli *et al*.^46,47^, so the discussions in this section will focus on the performance of the 16-layer AMR.

**Figure 3:**
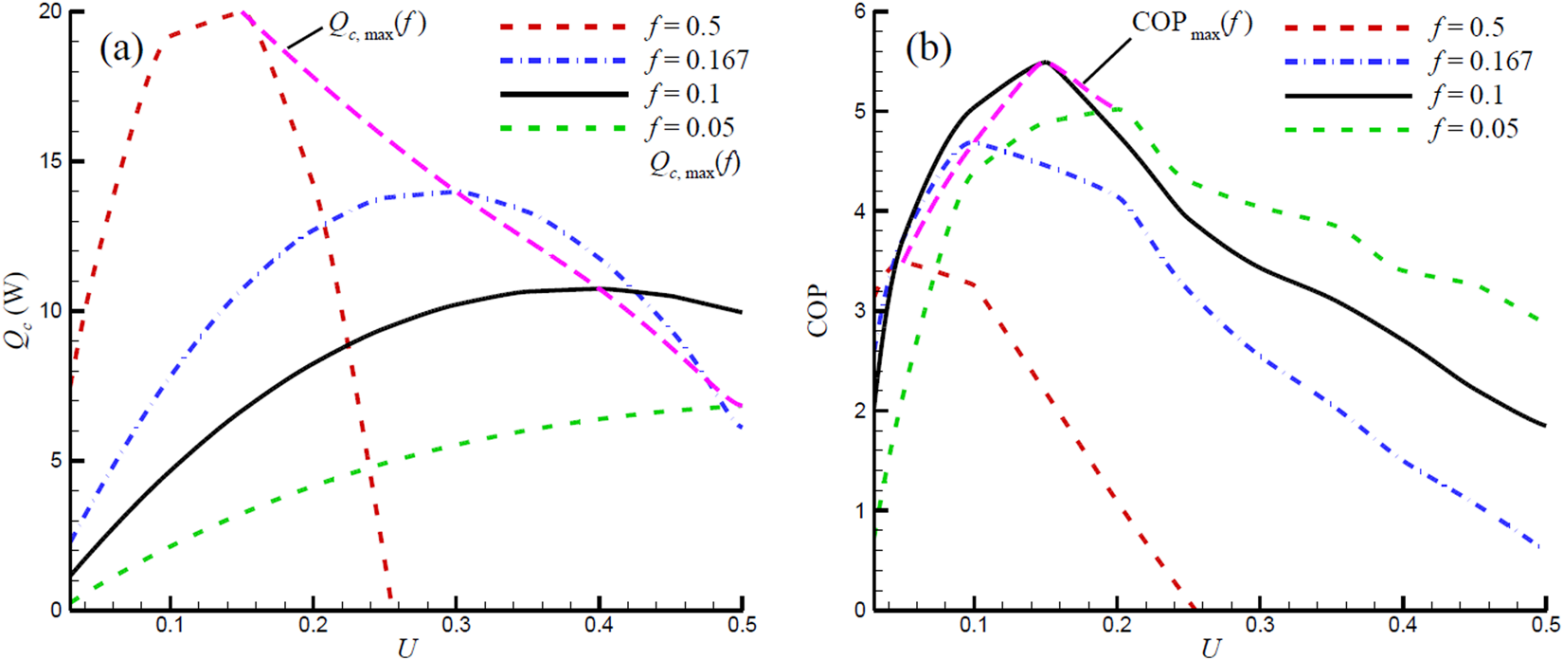
Cooling power and COP variations with utilization for different frequency.

With the configuration shown in Fig. 1, a series of simulations were conducted to investigate the effect of utilization *U* on the performance of the refrigerator. Figure 3(a) depicts the cooling power *Q*_*c*_ change with utilization at different operating frequencies. For a fixed frequency, the cooling power increases with *U* initially and then decreases after reaching a maximum value. When the utilization is low, the heat transfer fluid moves slowly and small quantity of low temperature fluid reaches the cold side. Therefore, only little cooling power is produced. On the other hand, when the fluid moves too quickly, the temperature profile of the AMR is disturbed, resulting in a loss of cooling power as well^48^. Note that the cooling power is negative, and not shown in this plot, when *U* > 0.255 and *f* = 0.5 Hz, indicating that the disturbed temperature profile leads to *T*_*f*_ (*x* = *L*) > *T*_*c*_ in the cooling processing and the refrigerator does not produce cooling effect under this condition. Consequently, for a 16-layer AMR running under a certain frequency, there exists a *U*, as shown in Fig. 3(a), that produces maximum cooling power. The corresponding *U* of the maximum cooling power increases as frequency decreases. It is because a higher *U* (e.g. fluid speed) is required to disturb the temperature profile in a lower frequency AMR. Figure 3(a) also indicates that the maximum cooling power increases with frequency. This occurs because increasing the operation frequency not only increases the rate of fluid motion, but also enhances the rate of entropy change (see Equation (1)) of the MCM to generate more cooling power.

Figure 3(b) illustrates the COP change with *U* at different operating frequencies. The COP at *f* = 0.5 and *U* > 0.255 is not shown here because it is less than zero due to the negative cooling power discussed above. Results indicate that when *f* = 0.5, the COP always decreases with *U* from 0.05 to 0.25. The trends of the COP of other frequencies are similar to the cooling power, i.e. there exists a utilization that results in a maximum COP. Figure 3(b) indicates that the maximum COP at *f* = 0.167, 0.1, and 0.05 Hz are 4.7 and 5.5 and 5.0 occurring at *U* = 0.1, 0.15, and 0.2 respectively, so the COP of the 16-layer AMR system reaches its maximum at frequency around 0.1 Hz. This is attributed to the competition between *Q*_*c*_ and ${\dot{W}}_{mag}$, both of which increase with as frequency increases. When the frequency is higher than 0.1 Hz, ${\dot{W}}_{mag}$ increases faster than *Q*_*c*_, leading to reduction of COP_max_ with increasing frequency. When frequency is less than 0.1 Hz, the increase of *Q*_*c*_ overcomes ${\dot{W}}_{mag}$. As a result, COP_max_ increases with increasing frequency. The corresponding *U* of the COP_max_ increases as the frequency decreases, although it is smaller than that of maximum cooling power at the same frequency. This is due to the mismatch between the maximum values of *Q*_*c*_ and ${\dot{W}}_{mag}$. Because of this mismatch, it is impossible to obtain the highest *Q*_*c*_ and COP in one configuration simultaneously. Therefore, after considering the priority of COP and comparing the 3 optimized point candidates, which are *f* = 0.167, *f* = 0.1 and *f* = 0.05 Hz at *U* = 0.1, *U* = 0.15, and *U* = 0.2, the point of *f* = 0.1 Hz and *U* = 0.15 was selected as the optimized working condition of the present 16-layer AMR since it provides the highest COP and an acceptable *Q*_*c*_. This frequency, *f* = 0.1 Hz, will be used in the discussions below.

### Performance comparison of the AMR with different number of layers

**Figure 4:**
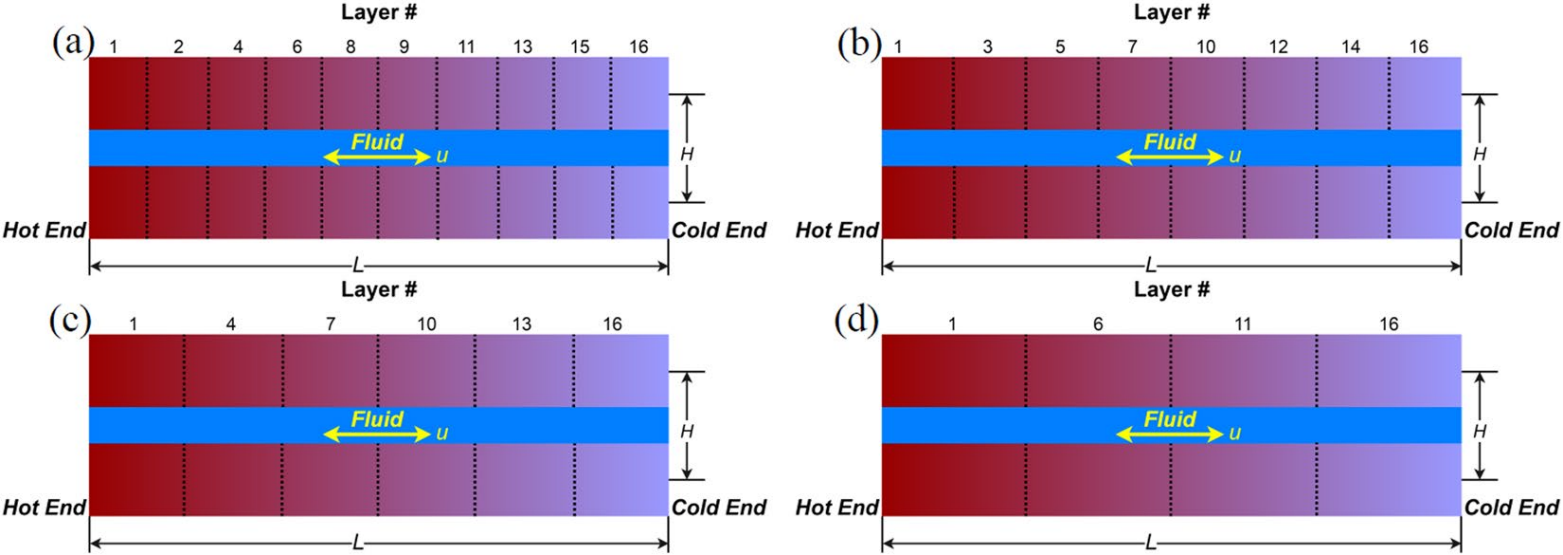
Schematic view of the 10-layer (**a**), 8-layer (**b**), 6-layer (**c**), and 4-layer (**d**) AMR.

Different regenerators with different number of layers (*N*_layer_) were investigated, including 4-layer, 6-layer, 8-layer, 10-layer and 16-layer AMRs at a frequency of 0.1 Hz determined from the performance analysis. Since the Curie temperatures of the MCM layers are different, various combinations of MCM layers were chosen to comprise the AMRs. The configurations of AMRs with different *N*_layer_ are depicted in Fig. 4. Note that all investigated AMRs had the same length, height, width, and porosity, regardless of their number of layers. The only difference among them was the length of each MCM layer. In Fig. 4, the layer # represents the layer number of the MCM in the 16-layer regenerator, e.g. in the 4-layer regenerator, Layer # 1, 6, 11 and 16 indicate that the materials are the same as the MCM Layers 1, 6, 11 and 16 in the 16-layer regenerator. Simulations of AMRs with different *N*_layer_ were conducted under the same conditions to compare their performances.

**Figure 5:**
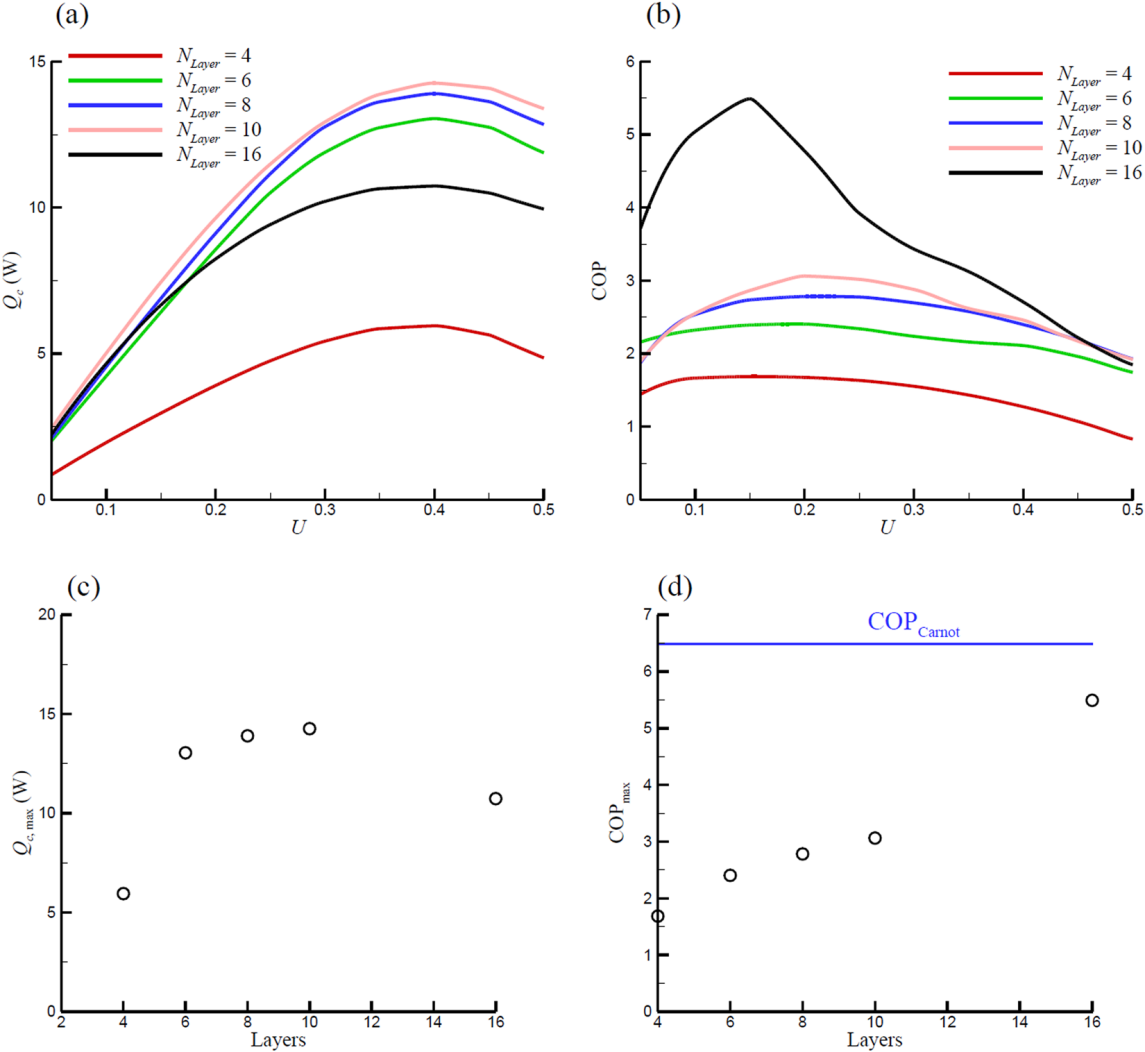
*Q*_*c*_ (**a**), COP (**b**), *Q*_*c*,max_ (**c**), and COP_max_ (**d**)variations with utilization for different *N*_*layer*_.

Figure 5(a) depicts the *Q*_*c*_ change with *U* for different AMRs with different *N*_layer_. As described above, for all the studied regenerators, *Q*_*c*_ increases to a maximum then decreases with *U*, generating a maximum cooling power (*Q*_*c*,max_) at about *U* = 0.4. As expected, the 4-layer AMR demonstrates the lowest cooling power, i.e the highest cooling power is less than 6 W from the 4-layer AMR. With the *N*_layer_ increasing from 6 to 10, the cooling power is improved significantly, which reaches as high as 14.3 W for the 10-layer AMR. To clearly illustrate the relationship between *Q*_*c*,max_ and number of layers in AMR, Fig. 5(c) depicts the *Q*_*c*,max_ as a function of the numbers of layers. Figure 5(c) shows that *Q*_*c*,max_ of the 6-layer AMR is less than the 8-layer one, while the 10-layer AMR provides the greatest *Q*_*c*,max_. Since the Curie temperatures of the MCM layers of AMRs fit the temperature distribution along the length of the regenerator, the shorter layer length leads to a narrower temperature span in one single layer of 10-layers AMR than AMR with less layers. This allows AMRs to take full advantage of the cooling ability of the individual MCM layers, leading to a higher cooling power. However, when *N*_layer_ = 16, the maximum cooling power drops. As shown in Fig. 5 (c), the *Q*_*c*,max_ of the 16-layer AMR is less than the 6-layer one. This is because going from 10-layer to 16-layer AMR, the arrangement of the MCM layers becomes denser. This means that the adjacent layers have only 2.5 or 2.25 K temperature difference for the 16-layer AMR, which may lead to a mismatch in the regenerator operating temperature. This mismatch in the regenerator operating temperature range in 16-layer AMR may not provide the optimal total entropy change comparing to the AMR with fewer layers (e.g. 10-layer), which has been reported by Govindappa *et al*.^28^. This phenomenon was also observed by Lei *et al*.^34^. Their results indicate that the cooling power does not monotonically increase with *N*_layer_. Since the properties of different layers in their simulation is identical (only shifting by Curies temperatures), it only occurs when the temperature span is low. However, due to the difference of entropy curve for every layer, the phenomenon observed in present work happens in a greater temperature span. Moreover, it implies that an optimal *N*_layer_ exists (here is *N*_layer_ = 10) for the AMRs in present work, that could provide the highest cooling power among the candidate options.

Figure 5(b) shows the COP change with *U* for AMRs with different *N*_layer_, which illustrates that the COP from the 4-layer regenerator is always less than 1.7 for any utilization. When *N*_layer_ is increased from 4 to 6, 8 and 10, improvement in COP in these regenerators is observed (COP_max_ from 1.6 for 4-layer to 3 for 10-layer) due to the cooling power enhancement of the AMR with more layers. However, the COP_max_ in these three regenerators never exceeds 3.1 as shown in Fig. 5(b). From the previous utilization discussions, it was concluded that a desynchronization exists between *Q*_*c*,max_ and COP_max_ as a function of *U*, i.e. a certain value of utilization cannot provide both the highest *Q*_*c*_ and the highest COP simultaneously. This trade-off between COP_max_ and *Q*_*c*,max_ also can be observed for the performance variance as a function of *N*_layer_. Figure 5(b) shows that although *N*_layer_ = 10 is the optimal number of layers for cooling power, it is not the optimum choice for COP; the16-layer AMR offers the highest COP (about 5.5) at *U* = 0.15. As discussed above, due to the competition between *Q*_*c*_ and ${\dot{W}}_{mag}$, the *U* for the COP_max_ is always less than the *U* for the *Q*_*c*,max_, so COP_max_ occurs at *U* = 0.15 or 0.2 for all the AMRs instead of *U* = 0.4, corresponding to *Q*_*c*,max_. It can be observed that in the range of *U* = 0.15 to 0.2, except the 4-layer AMR, the cooling powers of the different AMRs are very close. Therefore, the reason for the high COP_max_ of the 16-layer AMR can be attributed to the low ${\dot{W}}_{mag}$ in the 16-layer AMR due to the significant variation of MCM properties (e.g. entropy and specific heat) with temperature. It can be concluded that the combined effects of the mismatch of *Q*_*c*_ and ${\dot{W}}_{mag}$ as a function of *U* and the low ${\dot{W}}_{mag}$ in the 16-layer AMR lead to the highest COP_max_ from 16-layer AMR. Also, it is not feasible to obtain a highest *Q*_*c*_ and COP from an AMR simultaneously.

Figure 5(d) depicts the COP_max_ of different investigated AMRs with varying number of layers. It can be concluded that significant COP enhancement was achieved by utilizing the 16-layer AMR. As mentioned above, the COP of the 16-layer AMR can reach as high as 5.5, which amounts to 84.6% of the Carnot cycle COP.

### Optimization of the 16-layer parallel plates regenerator

Concerns over optimal distribution of MCM layers in multi-layer AMRs have stimulated studies to optimize the MCM layers^19,26,32^. It is essential to investigate the optimal distribution of the 16-layer parallel plates regenerator since it could provide guidance to achieve higher cooling power and COP. To accomplish this task, a DoE was employed to guide the parameters in numerical experiments and condense the essential number of numerical simulations. After the simulations were conducted following the DoE, a plot was generated to pick the cooling power and COP. Since the total length of the MCM layers is *L*, which is a constraint on the component proportions, a constrained mixture design was used to assess the influence of the length of every MCM layer on the overall regenerator performance^49^. Three levels of individual layers’ lengths were considered in present work, which are $\frac{15}{256}\,$ *L*, $\frac{23}{256}\,$ *L*, and $\frac{31}{256}\,$ *L*, while the MCM with uniform length is $\frac{16}{256}\,$ *L*. The details of the constrained design are shown in Table 2, of which the total number of runs is ${C}_{16+1}^{2}$+1 = 137. *L*_1_, *L*_2_, …, *L*_16_ denote the length of layer # 1, 2, …, 16 in Fig. 1(a), respectively. The first 16 runs set every one layer length as $\frac{31}{256}\,$ *L* in turn with other layers length $\frac{15}{256}\,$ *L*, while the runs 17 to 136 set the lengths of two layers as $\frac{23}{256}\,$ *L* each in turn with other layers length $\frac{15}{256}\,$ *L*. The last run is the reference run in which all layers have the same length, $\frac{16}{256}\,$ *L*.

**Table 2:** The lengths for 16 layers MCM in the DoE.

Run	*L*_1_/*L*	*L*_2_/*L*	*L*_3_/*L*	*L*_4_/*L*	*L*_5_/*L*	*L*_6_/*L*	*L*_7_/*L*	*L*_8_/*L*	*L*_9_/*L*	*L*_10_/*L*	*L*_11_/*L*	*L*_12_/*L*	*L*_13_/*L*	*L*_14_/*L*	*L*_15_/*L*	*L*_16_/*L*
1	**31/256**	15/256	15/256	15/256	15/256	15/256	15/256	15/256	15/256	15/256	15/256	15/256	15/256	15/256	15/256	15/256
2	15/256	**31/256**	15/256	15/256	15/256	15/256	15/256	15/256	15/256	15/256	15/256	15/256	15/256	15/256	15/256	15/256
15	15/256	15/256	15/256	15/256	15/256	15/256	15/256	15/256	15/256	15/256	15/256	15/256	15/256	15/256	**31/256**	15/256
16	15/256	15/256	15/256	15/256	15/256	15/256	15/256	15/256	15/256	15/256	15/256	15/256	15/256	15/256	15/256	**31/256**
17	**23/256**	**23/256**	15/256	15/256	15/256	15/256	15/256	15/256	15/256	15/256	15/256	15/256	15/256	15/256	15/256	15/256
18	**23/256**	15/256	**23/256**	15/256	15/256	15/256	15/256	15/256	15/256	15/256	15/256	15/256	15/256	15/256	15/256	15/256
30	**23/256**	15/256	15/256	15/256	15/256	15/256	15/256	15/256	15/256	15/256	15/256	15/256	15/256	15/256	**23/256**	15/256
31	**23/256**	15/256	15/256	15/256	15/256	15/256	15/256	15/256	15/256	15/256	15/256	15/256	15/256	15/256	15/256	**23/256**
32	15/256	**23/256**	**23/256**	15/256	15/256	15/256	15/256	15/256	15/256	15/256	15/256	15/256	15/256	15/256	15/256	15/256
33	15/256	**23/256**	15/256	**23/256**	15/256	15/256	15/256	15/256	15/256	15/256	15/256	15/256	15/256	15/256	15/256	15/256
44	15/256	**23/256**	15/256	15/256	15/256	15/256	15/256	15/256	15/256	15/256	15/256	15/256	15/256	15/256	**23/256**	15/256
45	15/256	**23/256**	15/256	15/256	15/256	15/256	15/256	15/256	15/256	15/256	15/256	15/256	15/256	15/256	15/256	**23/256**
134	15/256	15/256	15/256	15/256	15/256	15/256	15/256	15/256	15/256	15/256	15/256	15/256	15/256	**23/256**	**23/256**	15/256
135	15/256	15/256	15/256	15/256	15/256	15/256	15/256	15/256	15/256	15/256	15/256	15/256	15/256	**23/256**	15/256	**23/256**
136	15/256	15/256	15/256	15/256	15/256	15/256	15/256	15/256	15/256	15/256	15/256	15/256	15/256	15/256	**23/256**	**23/256**
137	16/256	16/256	16/256	16/256	16/256	16/256	16/256	16/256	16/256	16/256	16/256	16/256	16/256	16/256	16/256	16/256

**Figure 6:**
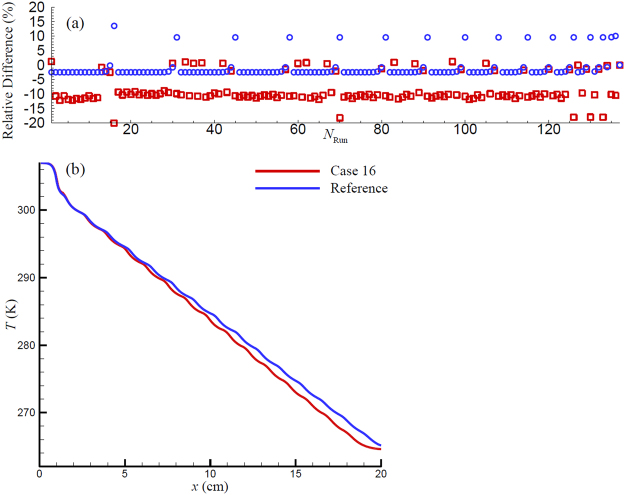
(**a**) Relative differences of Cooling power and COP for the 137 runs DoE comparing to the reference run. The circles and squares denote the results for Cooling power and COP respectively. (**b**) Temperature profiles for the case 16 and reference case along the regenerator at the end of cycle with the steady state has been reached.

Following the guidance of the DoE, a set of 137 simulations were conducted for *f* = 0.1 Hz and *U* = 0.15 obtained in Section 1, since the two parameters are the optimized working conditions for COP of all of the 137 runs. The cooling power and COP results from these simulations are compiled in Fig. 6(a), in which the squares and circles denote the results of COP and cooling power, respectively. The results are in the form of relative difference defined as (*R*_*i*_ − *R*_*ref*_)/*R*_*ref*_, where subscript *i* and *ref* indicate the *i*th run from DoE and the reference case, respectively, and *R* represents either *Q*_*c*_ or COP.

Figure 6(a) reveals that most of *Q*_*c*_ from the DoE cases are close to the reference case as shown by the negative relative difference with small absolute values. Only a few runs return a higher cooling power, which are more than a 10% increase from the reference case. By examining the DoE parameters, it was discovered that every high cooling power result corresponds to the cases with a longer layer# 16, e.g. case 16, 31, 45, 135 and 136 in Table 2. Note that layer# 16 is the last layer of MCM, which is located just next to the cold end of the regenerator, as shown in Fig. 1(a). Figure 6(b) depicts the temperature profiles along the MCM at the end of cooling cycle for case 16 and the reference case. It shows the MCM temperatures next to the hot end are very close between the two cases. However, next to the cold end, the temperature in the case 16 is much lower than the reference case, due to the longer layer# 16. Therefore, in the cooling cycle, a longer layer# 16 generates a lower temperature range next to the cold end as compared to the reference case, which is the reason for the 10% increase in the cooling power.

Moreover, Fig. 6(a) also presents the COP distribution of the DoE cases. It reveals that the values of COP of most of the DoE cases are about 10% less than the reference case. Considering that most of these cases yield smaller cooling power compared to the reference case, it can be concluded that the non-uniform layer lengths have a stronger effect on the magnetic work than on the cooling power, leading to higher drop of COP than cooling power. Figure 6(a) also reveals that when the non-uniform layer lengths are utilized in the 16-layer AMR, only trivial COP benefit is gained, i.e. the greatest COP enhancement from DoE cases is only 1.19% compared to the reference case. However, it can be identified from Fig. 6(a) that all of the cases with a longer layer# 16 result in a COP recession, e.g. the COP in case 16 is about 20% less than the reference case, while in case 31, a smaller increase in the length of layer 16 leads to an increase in *Q*_*c*_ that is as great as the decrease in COP (about 10% for both). This is due to the competition between ${\dot{W}}_{mag}$ and *Q*_*c*_: the longer layer# 16 not only enhances *Q*_*c*_, but also requires more power to overcome the increasing ${\dot{W}}_{mag}$. In the cases with a longer layer# 16, the increasing ${\dot{W}}_{mag}$ is more than *Q*_*c*_ enhancement, resulting in a lower COP output. Therefore, although the longer layer# 16 provides a higher cooling power, the dramatic COP drop eliminates them from the list of optimization.

The DoE results can be summarized as: (1) Most of non-uniform layer cases lead to a lower *Q*_*c*_ and a much lower COP compared to the reference case; (2) Although some cooling power benefits could be obtained from some cases with a longer layer# 16, all these cases result in a significant COP drop; (3) only a few combinations of non-uniform layer length are capable of providing COP benefits, which are no more than 1.2% compared to the reference case. Therefore, some optimization work may be required for the previous work with fewer layers MCM; however, this work suggests limited benefit of optimizing the length of every MCM layer for present 16-layer AMR case. This is because the Curie temperatures of the adjacent MCM layers of the 16-layer regenerator are very close to each other. As a result, there is no space to improve the COP.

## Conclusions

A room temperature magnetic refrigerator with a 16-layer parallel plates regenerator has been numerically investigated in present work. The property data of these 16 MCMs with different Curie temperatures was derived from experimental measurements provided by the material manufacturer. The Curie temperatures were from 268.4 K to 305.4 K with temperature steps 2.25 or 2.5 K. A 1D model was employed based on the previous single layer AMR model, in which a new method was developed to accelerate the model to make it executable for the multi-layer AMR model. Results indicate that the computational time of a 16-layer case is very close to the single-layer case using the new method, while, without the new method, the time required is almost 6 times that of the single-layer case.

Using the new model, performance analysis of the 16-layer AMR was conducted between 41 K temperature span and a maximum applied magnetic field of 1.5 T. It was found that for every frequency, there exists a maximum cooling power for the 16-layer AMR. The *Q*_*c*, max_ decreases as frequency increases. The same maximum COP with frequency variance is also observed except for the highest frequency case. The results reveal that around *f* = 0.1 Hz, the 16-layer AMR reaches its highest COP.

Additionally, a performance comparison between AMRs with different layers is provided in the present work. The results suggest that the AMR with a 10-layer regenerator offers the greatest cooling power. However, the AMR with a 16-layer regenerator can significantly enhance the system COP_max_, which could reach 84% COP of the Carnot cycle. These results guided an optimization investigation of layer length of the 16-layer AMR. A constrained mixture design, as a kind of Design of Experiments, was employed to generate a series of numerical experiments for the optimization. The design includes 137 simulations. According to the results, it is not essential to use a 16-layer AMR with non-uniform layer lengths, since it cannot find a significant COP enhancement by adjusting the layer lengths in DoE. This is explained because the Curie temperature between the adjacent layers is very close (2.25 or 2.5 K), and there is no room to improve COP by adjusting the space length of the layers.

### Data availability statement

All data generated or analysed during this study are included in this published article.
